# Outdoor walking: Mobile EEG dataset from walking during oddball task and walking synchronization task

**DOI:** 10.1016/j.dib.2022.108847

**Published:** 2022-12-23

**Authors:** Joanna E.M. Scanlon, Nadine S.J. Jacobsen, Marike C. Maack, Stefan Debener

**Affiliations:** a Neuropsychology Lab, Department of Psychology, University of Oldenburg, Germany; b Cluster of Excellence Hearing4all, University of Oldenburg, Oldenburg, Germany; c Center for Neurosensory Science and Systems, University of Oldenburg, Oldenburg, Germany; d Branch for Hearing, Speech and Audio Technology HSA, Fraunhofer Institute for Digital Media Technology IDMT, Oldenburg, Germany

**Keywords:** Mobile EEG, Attention, Gait, Walking, Social neuroscience, Interpersonal synchronization

## Abstract

This article describes a dataset from one standing and two outdoor walking tasks. Each task was performed by the same 18 participants twice, using foot accelerometers and two different EEG system configurations. The first task was a brief eyes open/eyes closed task. The second task was a six minute auditory oddball task performed in three conditions: Standing, walking alone and walking next to an experimenter. In the third task, the participants walked with the experimenter in three conditions: With their view of the experimenter blocked, walking naturally, and trying to synchronize their steps with the experimenter. During all walking conditions which included the experimenter, the experimenter walked following a headphone metronome to keep their steps consistent, also wearing a foot accelerometer. All tasks were performed twice on two separate days, using active electrode and passive electrode EEG configurations (Brain Products, GmbH). Data was used for Scanlon et al. (2021) and Scanlon et al. (2022), and could be used for learning about attention, walking mechanisms and social neuroscience.

Scanlon, J. E., Jacobsen, N. S. J., Maack, M. C., & Debener, S. (2021). Does the electrode amplification style matter? A comparison of active and passive EEG system configurations during standing and walking. *European Journal of Neuroscience, 54*(12), 8381–8395.

Scanlon, J. E. M., Jacobsen, N. S. J., Maack, M. C., & Debener, S. (2022). Stepping in time: Alpha‐mu and beta oscillations during a walking synchronization task. NeuroImage, 253, 119099.

**Specifications Table**

**Table:** 

Subject	Neuroscience: Cognitive
Specific subject area	Cognitive neuroscience of gait, attention and interpersonal synchrony
Type of data	EEG in BIDS format
How data were acquired	**Instruments:** Mobile EEG using two configurations (active & passive electrodes), 2 accelerometer sensors (data not included, but step timing is marked with data triggers). **Make and model and of the instruments used:** actiCAP Slim, EasyCap GmbH, Brain Products GmbH, EasyCap GmbH, Brain Products GmbH, 2 LiveAmp mobile EEG amplifiers (Brain Products GmbH), Faros 180° eMotion (Mega Electronics, 2017), Dell (Latitude 5289) Ultrabook
Data format	Raw EEG data
Parameters for data collection	Participants were recruited from the University of Oldenburg and were required to have no neurological problems, etc. They needed to be fit enough to be standing & walking for about an hour (with breaks). Data were only collected on days with relatively calm weather between ∼15 °C and 35 °C. Data were collected in an outdoor roofed area, so moderate rain did not prevent recording.
Description of data collection	Participants performed three tasks outdoors while wearing 2 types of mobile EEG and an accelerometer on their right foot. The first task was a brief eyes open/eyes closed task. The second task was an auditory oddball task performed in three conditions: Standing, walking alone and walking next to an experimenter (who also wore an accelerometer). In the third task, the participants walked with the experimenter in three conditions: With their view of the experimenter blocked, walking naturally, and trying to synchronize their steps with the experimenter.
Data source location	Institution: Carl von Ossietzky Universität Oldenburg City/Town/Region: Oldenburg, Lower Saxony Country: Germany Latitude and longitude (and GPS coordinates, if possible) for collected samples/data: 53.147118056519375, 8.178847795546195
Data accessibility	Data is hosted on a public repository Repository name: Openneuro.org Data identification number: ds004033 Direct URL to data: doi:10.18112/openneuro.ds004033.v1.0.0
Related research article	Scanlon, J. E., Jacobsen, N. S. J., Maack, M. C., & Debener, S. (2021). Does the electrode amplification style matter? A comparison of active and passive EEG system configurations during standing and walking. *European Journal of Neuroscience, 54*(12), 8381–8395. Scanlon, J. E. M., Jacobsen, N. S. J., Maack, M. C., & Debener, S. (2022). Stepping in time: Alpha‐mu and beta oscillations during a walking synchronization task. NeuroImage, 253, 119099. Note: two projects were done with this data because it contains two separate tasks

## Value of the Data

• These data are useful for learning about MoBI (Mobile brain/body imaging) signal processing and hardware, walking, attention, and interpersonal synchronization during gait, because they contain two mobile EEG tasks recorded outdoors, using two different EEG electrode configurations.
• This dataset can benefit cognitive neuroscientists who study the brain during gait, as well as neurorehabilitation patients, because EEG recorded during walking can be used to learn about gait mechanisms in the brain.
• These data can be used for various comparisons between conditions, and also can be reanalyzed using new methods such as source localization.

## 1 Data Description

This dataset contains data from 18 subjects, performing 3 tasks on two separate days. This includes EEG data using active and passive electrodes, as well as step timing data from both the participant and the experimenter. The data are documented in the BIDS format, which describes all timing triggers and all important information for the experiment. For each participant, there is one folder for active and one for passive electrodes. Data files within these folders include .set and .fdt files which include 64 channels of EEG data, 3 channels from an accelerometer within the amplifiers, as well as event triggers corresponding to step detection from the experimenter and participant. Additionally there are files which list information for all EEG channels (‘channels.tsv’), electrode channel locations (‘electrodes.tsv’) and the coordinate system (‘coordsystem.tsv’) used.

All triggers in the experiment event structure are included and described. This includes those for instructions, onset of the eyes open/eyes closed task, oddball triggers differentiated by condition, and onset of the walking task conditions. Additionally, triggers are included for heel-strike and toe-off events found in the accelerometer data in task 3 (see *Accelerometer synchronization and step triggers*), for both the experimenter and participant, differentiated by condition. A full description of these triggers is included in the ‘events.json’ files, with timing information contained in the ‘events.tsv’ files for each participant and session.

## 2 Experimental Design, Materials and Methods

### 2.1 Participants

Twenty-six participants were recruited for the study, through the Oldenburg University website. Data were removed from seven participants from the study due to technical issues during data collection. This was mainly due to problems in the amplifier's wireless connection, which have since been addressed with the manufacturer and solved. Data from one participant were removed due to issues scheduling the second appointment. For the final analysis, this left 18 participants (mean age  = 24; age range: 20–28; eight female). Participants were all right-handed and had no history of neurological or psychiatric problems. For participating in the study, participants received an honorarium of 10 €/hour. Experimental procedures were approved by the Oldenburg University ethics committee.

### 2.2 Materials

**Fig. 1:**
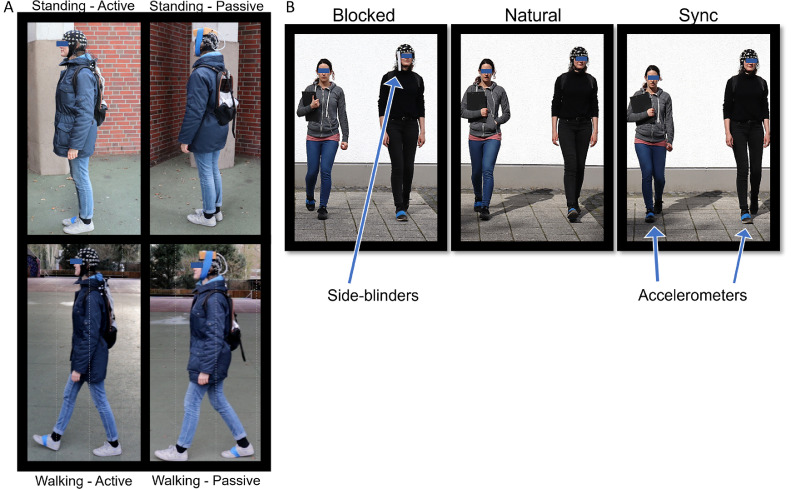
Task conditions and set-up. (a): Task 2 (from [5]): The conditions used during the oddball task (task 2; staged image): Standing with active electrodes, standing with passive electrodes, walking alone with active electrodes, walking alone with passive electrodes. The same standing area was used for the standing eyes open/eyes closed task (task 1). (b) Main setup components of the three walking conditions in task 3 (staged images; from [6]): walking with view of the experimenter blocked (left), walking naturally (middle), and walking while trying to synchronize steps with the experimenter (right). The *natural* condition also depicts an identical walking configuration to the *walking together* oddball condition in task 2.

Participants were asked to come in twice for two sessions to record with each of two electrode configuration types. Appointments were approximately 3–15 (Mean (M) = 6) days apart. Order of electrode type was counterbalanced across participants. Recording time of day was within the same hour for 10 participants, and in cases when the same time could not be scheduled for both sessions, earlier/later times were counterbalanced according to electrode type. For each experiment session, participants were fitted with an EEG cap with either passive (EasyCap GmbH, Brain Products GmbH) or active electrodes (actiCAP, EasyCap GmbH, Brain Products GmbH), each with identical 64 electrode layouts. EEG and accelerometer data were collected with a 500 Hz sampling rate. Reference and ground electrodes were embedded at the FCz and AFz (10–20 system) locations in the cap, respectively. For all sessions, the same two Brain Products LiveAmp amplifiers, which consisted of one amplifier for channels 1–32 and one for channels 33–64 (which were never switched), were used. A Faros 180° eMotion (Mega Electronics, 2017) accelerometer sensor was fixed to the right foot of each participant and the experimenter using elastic tape. The auditory oddball task was presented on a Dell (Latitude 5289) Ultrabook and ear-phones (Sony MDR-E9LP) using NBS (Neurobehavioral Systems) Presentation. Data were collected using LSL (Lab Streaming Layer) software to time-synchronously collect both the EEG channel data and the event markers from NBS Presentation, using the same Ultrabook computer. Accelerometer and EEG data were synchronized using TTL pulses sent from a sync-box. The sync-box is a device similar to the TriggerBox (Brain Products GmbH), which sent TTL pulses into the EEG and accelerometer data (see *Accelerometer synchronization and step triggers*). During sessions with active electrodes, the LiveAmp and Ultrabook were placed into a small backpack which was customized for the ventilation of the computer. All loose wires were tucked into the backpack in order to minimize wire movements. During sessions with passive electrodes, the LiveAmp was fixed to the top of the participant's head using elastic tape and a customized sponge that included holes to avoid any direct physical pressure on underlying electrodes (Fig. 1a), and the ultrabook was also placed inside the backpack.

The experiment took place in an outdoor roofed basketball arena at the Oldenburg University campus. Participants had the experimental equipment applied at the lab, and then walked 5 mins away to perform the experiments at the outdoor arena. This allowed the experimenter to build rapport with the participant and served as a short ‘warm up’ for the walking tasks. Pylons were placed in a rectangular formation around the whole arena to guide the experiment and occasionally were adjusted to avoid rain or excessive sunlight.

### 2.3 Accelerometer Synchronization and Step Triggers

At the beginning and end of each recording, synchronization reference signals were sent into the EEG and accelerometer data. To do this, the LiveAmp and Faros sensors were plugged into the sync-box at the start and end of each data recording, which sent TTL pulses into the data at the same time. This follows the rational from [1], who demonstrated that MoBI-quality synchronization can be attained by marking data with a distinctive signal at the beginning and end of a recording and later using these signals to align the data. These distinctive signals can also be used to test synchronization quality and to troubleshoot issues relating to data synchronization. Custom MATLAB scripts were used to offline prune the data streams of accelerometer and EEG data into one synchronized data file. Acceleration data (in mg's) from the right foot of the experimenter and participant were detrended and filtered with a low-pass filter of 30 Hz (Butterworth; order 2). Custom algorithms (similar to [8] and [5]), using the Matlab function *findpeaks* were implemented to find heel-strike (HS) and toe-off (TO) events in the accelerometer data, and mark these timepoints (after a starting countdown) as events in each dataset. Accelerometer data was then removed from the datasets, to avoid complications with further analyses such as ICA. All MATLAB code from these steps, as well as all analyses for [6] is available at https://github.com/jscanlon275/jos_sync2.

### 2.4 Experiment Procedure

The whole experiment included three separate tasks. The first task was an eyes open/eyes closed task, in which the participants stood next to a wall (approximately 2 m away) for one minute in two conditions: with their eyes open or eyes closed. This procedure was repeated twice (2 min each condition) and order was counterbalanced. The second task was a 5–6 min auditory oddball task performed in three conditions: *Standing, walking alone* and *walking together* next to an experimenter (main design based on [2]). During the *standing* condition, participants stood with their eyes open, staring at a brick wall. During the *walking alone* condition, participants walked at their own pace around the arena in a clockwise direction, following pylons as a guide. They were encouraged to keep a slow pace in order to avoid aerobic effects. During the *walking together* condition, participants walked with the experimenter on their right side. The experimenter kept a constant pace by using a headphone metronome (see last paragraph for details). These 3 conditions were repeated twice. During the oddball task, participants were asked to silently count the number of deviant tones, and report the number to the experimenter at the end of each condition block (behavioural response data not included). Each block of the oddball task included 280 trials (with 15% deviants) plus an additional 1–30 extra trials at the end. The number of these extra trials was randomly selected for each block, in order for the number of deviants to be unpredictable between blocks. Additionally, the task was programmed so that two deviant tones were never played subsequently. The tones were 1000 and 800 Hz, and their standard (frequent) or deviant status was counterbalanced across participants. The inter-trial interval varied randomly between 500 and 1500 ms (125 ms uniform distribution). This high variability was chosen in order to avoid a rhythm with which participants could synchronize their steps. Each block lasted approximately 5–6 min and was repeated twice for each condition.

In the third task, the participants walked for 6 min clockwise around the arena with the experimenter (to their right) in three conditions: With their view of the experimenter blocked (*blocked*), walking naturally (*natural*), and while trying to synchronize their steps with the experimenter (*sync*). These conditions were counterbalanced, each lasting six minutes (main design based on [3], [4]). In the *blocked* condition, participants’ view of the experimenter was blocked, using ‘side-blinder’ glasses, similar to [7]. The side-blinders consisted of lensless plastic glasses, with laminated paper rectangles (7 cm x 20 cm; Fig. 1A) attached to the sides to block participants’ peripheral view. In the *natural* condition, participants simply walked next to the experimenter with no specific instructions about walking in relation to the experimenter. In the *sync* condition, the participants were asked to intentionally synchronize their steps with the experimenter. This meant that they tried to have their left foot forward when the experimenter's left foot was forward, and vice versa. Participants were asked to avoid gross head movements, and only use peripheral vision to see the experimenter. The participant's sound environment was not heavily controlled, as this study was focused on manipulating visual information. However, the experimenter avoided wearing noisy clothing during the experiment such as noisy shoes that ‘clicked’ or clothing that ‘swished’ as she walked. The arena had ambient noise from the nearby street which included passing cars, people talking, and some construction. This somewhat muffled the sound of both individuals’ footsteps. A beep sound signaled the end of each walking condition.

During all walking conditions which included the experimenter, the experimenter used a metronome app (Buluobang Inc.) on their phone, which played through headphones at 90 bpm. They aligned their stepping pattern to this rhythm, stepping down with each beat, in order to ensure that they stepped consistently throughout the conditions. The experimenter was also wearing a foot accelerometer on their right foot throughout the experiment, which allowed us to later test relational walking patterns between the experimenter and participant [6]. Order of conditions within each task, as well as oddball standard/deviant tones were counterbalanced across participants. Participants were then asked to walk in a clockwise direction around the arena (Fig. 1). At the beginning of each walking (and standing) condition, an audio countdown from 10 was played. This allowed the experimenter and participant to get into a comfortable walking pattern before starting the condition and get ready for the task to start. Further descriptions of the analysis of this data can be found in [5], [6].

## Ethics Statement

Informed consent for the study was obtained from all subjects on each testing day. The Oldenburg University ethics committee approved the experimental procedures (permit number: 2019–001).

## CRediT authorship contribution statement

**Joanna E.M. Scanlon:** Conceptualization, Methodology, Formal analysis, Writing – original draft, Visualization, Funding acquisition. **Nadine S.J. Jacobsen:** Conceptualization, Methodology, Software, Validation, Writing – review & editing. **Marike C. Maack:** Investigation, Conceptualization. **Stefan Debener:** Supervision, Conceptualization, Writing – review & editing, Resources, Funding acquisition.

## Declaration of Competing Interest

The authors declare that they have no known competing financial interests or personal relationships which have or could be perceived to have influenced the work reported in this article.

## Data Availability

Electrode walking study (Original data) (OpenNeuro.org).
